# 

**Published:** 1851-01

**Authors:** 


### TO READERS AND CORRESPONDENTS.

Communications intended for publication should be directed to Dr. C A. Harris, editor of the American Journal of Dental Science, Baltimore, Md. But all letters relating to the business of the Journal, or containing remittances in money, should be directed to Drs. C. A. Harris and A. A. Blandy, as the last named gentleman, who is associated with Dr. H. in practice, will have the management of the *business* connected with the publication.

The following Journals and Publications have been received.

Review of Chemistry for Students, adapted to the courses as taught in the principal Medical Schools in the United States. By John G. Murphy, M. D. From the publishers, Messrs. Lindsay & Blakiston, Philadelphia.

A Treatise on the Diseases and Surgical Operations of the Mouth, and Parts Adjacent; with notes of interesting cases, ancient and modern. Translated from the French of M. Jourdain, Dentist and Member of the College of Surgery. From the Publishers, Messrs. Lindsay & Blakiston, Philadelphia.

Proceedings of the American Philosopical Society, vol. v. No. 45, April and December, 1850.

An Introductoɹy Lecture, delivered at the Opening of the thirty-first Session, of the Medical College of Ohio, November, 1850. By John Bell, M. D., Professor of the Theory and Practice of Medicine, &c. &c.

The American Journal of the Medical Sciences. Edited by Isaac Hays, M. D., October, 1850.

The London Medical Gazette, October, 1850.

The Boston Medical and Surgical Journal. Edited by J. V. C. Smith, M. D., October, November and December, 1850.

The British and Foreign Medico-Chirurgical Review, October, 1850.

The New York Dental Recorder. Edited by C C. Allen, M. D., Dentist. November and December, 1850.

The Dental Register of the West. Edited by James Taylor, M. D. D. D. S., October, 1850. The July number has not been received. Will the Editor do us the favor of sending it.

The Dental News Letter, October, 1850.

The London Medical Times, October, 1850.

### *To Readers and Correspondents.*

The Buffalo Medical Journal. Edited by Austin Flint, M. D., November and December, 1850.

The Provincial Medical and Surgical Journal. Edited by W. H. Rankin, M. D., and J. H. Walsh, Esq., September and October, 1850.

The Dublin Medical Press, October, November and December, 1850.

The British, American, Medical and Physical Journal. Edited by Archibald Hall, M. D., October and December, 1850. The November No. has not been received; will the editor do us the favor to send it.

The Medical Examiner. Edited by F. G. Smith, M. D., November and December, 1850.

The Charleston Medical Journal and Review. Edited by D. J. Cain, M. D., and F. Peyrel Porcher, M. D., October and November, 1850.

The New York Journal of Medicine, and the Collateral Sciences. Edited by S. S. Purple, M. D., October and November, 1850.

The Southern Medical and Surgical Journal. Edited by J. P. Garvin, M. D., November and December, 1850.

The North-Western Medical and Surgical Journal. Edited by J. Evans, M. D., and E. G. Meek, M. D., September and October, 1850.

The New York Medical Gazette, and Journal of Health. Edited by D. Meredeth Reese, M. D., LL. D., November and December, 1850.

The Western Journal of Medicine and Surgery. Edited by L. P. Yandell, M. D., and T. S. Bell, M. D., October, November and December, 1850.

The St. Louis Medical and Surgical Journal. Edited by M. L. Linton, M. D., J. S. Moore, M. D., Wm. M. McPheeters, M. D., and J. B. Johnson, M. D., December, 1850.

The Ohio Medical and Surgical Journal. Edited by S. Hanbury Smith, M. D., October and November, 1850.

The Western Lancet and Hospital Reporter. Edited by L. M. Lawson, M. D., and George Mendenhall, M. D., October, November and December, 1850.

The New Hampshire Journal of Medicine. Edited by Edward H. Parker, A. M., M. D., September, October November and December, 1850.

The New Jersey Medical Reporter, and Transactions of the New Jersey Medical Society. Edited by Joseph Parrish, M. D., October, 1850.

The New York Register of Medicine and Pharmacy. Edited by C. D. Griswold, M. D., November and December, 1850.

The London Lancet, Journal of British and Foreign Medical, Surgical and Chemical Sciences, Criticism, Literature and News. Editor, Thos. Wakeley, M. D., for London: Sub-Editors, J. H. Bennet, M. D., and T. Wakeley, M. R. C. S. E., October, November and December, 1850.

The Dental Messenger and Lancaster Annual Visitor, for 1850. By John McCalla, D. D. S., Lancaster, Pa.

First Annual Announcement of the Transylvania School of Dental Surgery. Session 1850-'51.

The article from Dr. S. P. Hullihen, on a new kind of Artificial Palate, &c., was received too late for publication in the present number. It shall appear in the next. We also received an article, "A Test of Levett's Patent Enamel, and its Discovery," from Dr. C. T. Cushman, too late for insertion in this number.

G. R. D. is informed that his communication is of too miscellaneous a character for publication in the Journal. If he will embody that portion of it which relates to the best means for the elevation of the dignity of the dental profession, in a paper by itself, we will be most happy to give it a place in our periodical.

*Cuspidatus,* although seasoned with an abundance of spice, is too pointed to do good; we must, therefore, decline publishing it. We would, however, be most happy to receive an article from the author on some other subject.

The communication from C. R. has been received, and will receive attention at our earliest leisure.

---

### CONTENTS.

*To Subscribers.*—On the 1st of February we shall send bills to our delinquent subscribers. But we trust that all will thus save themselves a postage and ourselves a tedious amount of unnecessary labor, by immediately forwarding the amount, which will be promptly credited to their respective names; and as we intend to publish, in the April number, a list of those who have paid, we beg all our friends and subscribers to swell the list, not forgetting that, for the faithful and successful completion of our Journal, it must be sustained in this most necessary and potent manner. It is too near a fact, that most medical journals generally languish, fail and cease to be, from the want of prompt and well-directed support in this particular. Subscribers too often acknowledge the duty all are under of sustaining this great bulwark of science and professional advancement, but think, by the mere addition of their names, that this is all accomplished. But we would like to feel and know that our Journal is exempt from this great drawback and serious source of inconvenience. To those who have already remitted, we would return our most sincere thanks.

